# Trends of Underweight and Overweight/Obesity Among Japanese Schoolchildren From 2003 to 2012, Defined by Body Mass Index and Percentage Overweight Cutoffs

**DOI:** 10.2188/jea.JE20140144

**Published:** 2015-07-05

**Authors:** Takako Shirasawa, Hirotaka Ochiai, Hinako Nanri, Rimei Nishimura, Tadahiro Ohtsu, Hiromi Hoshino, Naoko Tajima, Akatsuki Kokaze

**Affiliations:** 1 Department of Public Health, Showa University School of Medicine, Tokyo, Japan; 2 Division of Diabetes, Metabolism and Endocrinology, Department of Internal Medicine, Jikei University School of Medicine, Tokyo, Japan; 3 Jikei University School of Medicine, Tokyo, Japan

**Keywords:** body mass index, percentage overweight, schoolchildren, secular trends, Japanese

## Abstract

**Background:**

We investigated the prevalence and trends of underweight and overweight/obesity in a population-based sample of Japanese schoolchildren from 2003 to 2012, defined by body mass index (BMI) and percentage overweight (POW).

**Methods:**

Subjects comprised fourth and seventh graders from the town of Ina, Japan, from 2003 to 2012. The height and weight of each subject were measured. Children were classified as underweight, normal weight, or overweight/obese using two criteria: BMI cutoff points proposed by the International Obesity Task Force and cutoffs based on POW in Japan.

**Results:**

Data from 4367 fourth graders and 3724 seventh graders were analyzed. The prevalence of underweight and overweight as defined by POW criteria were lower than those based on BMI criteria. There was a decrease in the prevalence of overweight among fourth-grade boys and girls and seventh-grade girls according to BMI; this decrease was also observed when POW criteria were used for the definition of overweight.

**Conclusions:**

The prevalence and trends of both underweight and overweight as defined by POW were underestimated among Japanese schoolchildren compared to those determined using BMI. The results of this study also suggest that trends in underweight and overweight/obesity using POW criteria are similar to those based on BMI criteria among schoolchildren in Japan.

## INTRODUCTION

Childhood overweight and obesity have increased dramatically in economically developed countries and in urbanized populations.^1^^,^^2^ A previous study showed that approximately half of overweight adolescents and over one third of overweight children became obese in adulthood, and that adiposity in childhood and adolescence influenced adult mortality and morbidity.^3^ Therefore, childhood obesity and overweight should be closely monitored for public health policy reasons.

In addition to childhood overweight and obesity, thinness among children has been reported to be associated with health problems. Previous studies indicated trends of unhealthy body image and dieting behaviors among pre-adolescents and adolescents in Japan, especially in girls.^4^^,^^5^ Moreover, thinness among adolescent girls has been reported to cause a number of additional health problems including eating disorders,^6^ secondary amenorrhea,^7^ and low bone density.^8^ Therefore, it is important from a public health standpoint to monitor underweight among children.

It is necessary to define underweight, overweight, and obesity in order to monitor and compare trends. Recently, the International Obesity Task Force (IOTF) proposed an international body mass index (BMI) reference standard to define underweight, overweight, and obesity among children and adolescents.^9^^–^^11^ Thus, current trends of underweight and overweight among children using these BMI cutoffs have been reported from many countries.^12^^–^^15^ In Japan however, cutoffs for schoolchildren have been based on percentage overweight (POW) rather than BMI.^16^ Although overall trends in BMI^17^ and the prevalence of overweight based on BMI cutoff points^18^ among schoolchildren have been reported in population-based studies, there have been no studies examining the secular trends of underweight and overweight over the last decade in Japan, as far as we know. In addition, a recent study showed that controversy remains regarding the selection of international standards for defining underweight and overweight in children.^19^ Thus, it is very important to investigate the trends of underweight, overweight, and obesity using both BMI and POW.

Accordingly, the aim of the present population-based study was to investigate the prevalence and trends of underweight and overweight/obesity among schoolchildren in Japan from 2003 to 2012, defined by BMI and POW cutoffs.

## METHODS

This study was conducted in the town of Ina, Japan, which is located in the southern part of Saitama, within a 40-km radius from the center of Tokyo.^20^ Since 1994, Ina has implemented a unique health promotion program to prevent childhood lifestyle-related diseases, in addition to the national annual health checkups conducted in accordance with the Japanese School Health Law.

Of the fourth and seventh graders who underwent regular health checkups between 2003 and 2012, those who volunteered to participate in the health promotion program also underwent a blood test, physical examinations, and a questionnaire survey. Although there have been previous studies regarding Ina’s health promotion program,^17^^,^^21^ there are no reports of secular trends in underweight and overweight. Therefore, the present study was conducted as a part of this program.

### Subjects

The subjects of this study comprised all fourth graders (aged 9–10 years) and seventh graders (aged 12–13 years) from town-run schools in Ina between 2003 and 2012. Written informed consent was obtained from the parent or guardian of each subject. The study protocol was approved by the Medical Ethics Committee of Showa University School of Medicine.

### Measurements

The height and weight of each child were measured at each school. For the measurements, all children wore light clothing but removed their shoes and socks, after which height and weight were measured in increments of 0.1 cm and 0.1 kg, respectively. The same examination protocol was used throughout the study period to ensure uniformity and precision of assessment. BMI was calculated as body weight (kg) divided by the height squared (m^2^).

### Definitions

Children were classified as underweight, normal weight, or overweight/obese using the following two criteria: BMI cutoff points proposed by IOTF^9^^–^^11^ and POW cutoff points proposed by the Ministry of Education, Culture, Sports, Science and Technology (MEXT) in Japan.^22^

BMI cutoff points for childhood underweight, overweight, and obesity were determined by the age- and sex-specific cutoff points linked to the BMI values of 18.5, 25, and 30 at age 18.^9^^–^^11^ In this study, obesity was included in overweight. Children who were not underweight or overweight were regarded as normal weight.

According to POW criteria, which are based on the age- and sex-specific standard body weight for their height, children with POW ≥ +20% were classified as overweight and those with POW ≤ −20% were classified as underweight. Children with POW between these cutoffs were classified as normal weight.^22^

### Statistical analysis

The number of subjects who were included as fourth graders during 2003–2009 and seventh graders during 2006–2012 was calculated. Data were analyzed separately by each grade and sex. The normality of distribution was tested for each variable. Characteristics of boys and girls were compared using the chi-square test or the unpaired *t* test. The prevalence of underweight, normal weight, and overweight during the study period was calculated using BMI and POW criteria by sex for each grade. The prevalence of underweight and overweight was analyzed using a linear regression model for trend. A *P* value of <0.05 was considered statistically significant. All data were analyzed using SPSS statistics 20.0 (IBM, Chicago, IL, USA).

## RESULTS

Of 4395 fourth graders and 3770 seventh graders, 24 and 44 children were excluded from the analysis because of refusal to participate or school absence, respectively. The rate of participation for the fourth and seventh graders was 99.5% and 98.8%, respectively. Eventually, data on sex, age, height, and weight from 4367 fourth graders and 3724 seventh graders were analyzed. Almost all fourth graders during 2003 to 2009 (*n* = 2862) were among the seventh graders during 2006 to 2012 (*n* = 2601).

Table shows the characteristics of the participants: 4367 children were fourth graders (2269 boys and 2098 girls), and 3724 children were seventh graders (1910 boys and 1814 girls), with mean ages of 9.3 and 12.2 years, respectively. BMI was significantly higher in fourth-grade boys than fourth-grade girls (*P* < 0.001), whereas it did not significantly differ between sexes in seventh graders (*P* = 0.290). According to BMI criteria, the prevalence of underweight in fourth-grade boys and girls was 8.9% and 12.0%, respectively, while in seventh-grade boys and girls it was 8.3% and 10.7%, respectively. The prevalence of overweight was 15.1% and 11.6% in fourth-grade boys and girls, respectively, while it was 14.3% and 11.2% in seventh-grade boys and girls, respectively. When using POW criteria, the prevalence of underweight and overweight was lower than that calculated using the BMI criteria, regardless of sex and grade.

**Table.** Characteristics of study subjects by grade and sex

		Fourth graders			Seventh graders		
	
		Boys (*n* = 2269)	Girls (*n* = 2098)	*P*-value	Boys (*n* = 1910)	Girls (*n* = 1814)	*P*-value
Age (years)	Mean (SD)	9.3 (0.4)	9.3 (0.4)	0.560	12.2 (0.4)	12.2 (0.4)	0.697
Height (cm)	Mean (SD)	134.4 (5.7)	134.4 (6.3)	0.813	154.0 (8.2)	152.5 (6.0)	<0.001
Weight (kg)	Mean (SD)	31.0 (6.3)	30.2 (6.0)	<0.001	44.5 (9.9)	43.7 (8.0)	0.010
BMI (kg/m^2^)	Mean (SD)	17.1 (2.6)	16.6 (2.3)	<0.001	18.6 (3.0)	18.7 (2.8)	0.290
	Median (IQR: Q3 to Q1)	16.5 (18.1 to 15.3)	16.2 (17.7 to 15.1)		17.9 (19.7 to 16.6)	18.3 (20.0 to 16.8)	
	Range (Max to Min)	23.6 (35.5 to 11.9)	18.9 (30.9 to 12.0)		25.5 (38.3 to 12.9)	24.0 (36.6 to 12.6)	
POW (%)	Mean (SD)	0.2 (14.1)	−1.0 (13.0)	0.003	−0.8 (15.4)	−2.3 (14.1)	0.001
	Median (IQR: Q3 to Q1)	−2.5 (6.3 to −9.2)	−3.5 (5.3 to −9.8)		−4.1 (4.5 to −10.6)	−4.6 (4.4 to −11.4)	
	Range (Max to Min)	119.1 (91.2 to −27.9)	100.4 (73.0 to −27.5)		132.9 (100.6 to −32.3)	116.0 (85.6 to −30.4)	
Criteria							
BMI^a^	Underweight (%)	8.9	12.0	<0.001	8.3	10.7	0.002
	Normal weight (%)	76.0	76.4		77.4	78.1	
	Overweight (%)	15.1	11.6		14.3	11.2	
POW^b^	Underweight (%)	2.0	2.1	0.152	3.3	6.2	<0.001
	Normal weight (%)	89.6	90.9		87.0	86.8	
	Overweight (%)	8.5	6.9		9.7	7.0	

BMI, body mass index; IQR, interquartile range; Max to Min, maximum to minimum; Q3 to Q1, 75th percentile to 25th percentile; SD, standard deviation.

^a^BMI cutoff points as defined by the International Obesity Task Force.

^b^POW (percentage of overweight) as defined by the Japanese Ministry of Education, Culture, Sports, Science and Technology.

The unpaired *t* test and chi-square test were used to compare characteristics between boys and girls.

**Figure 1.**
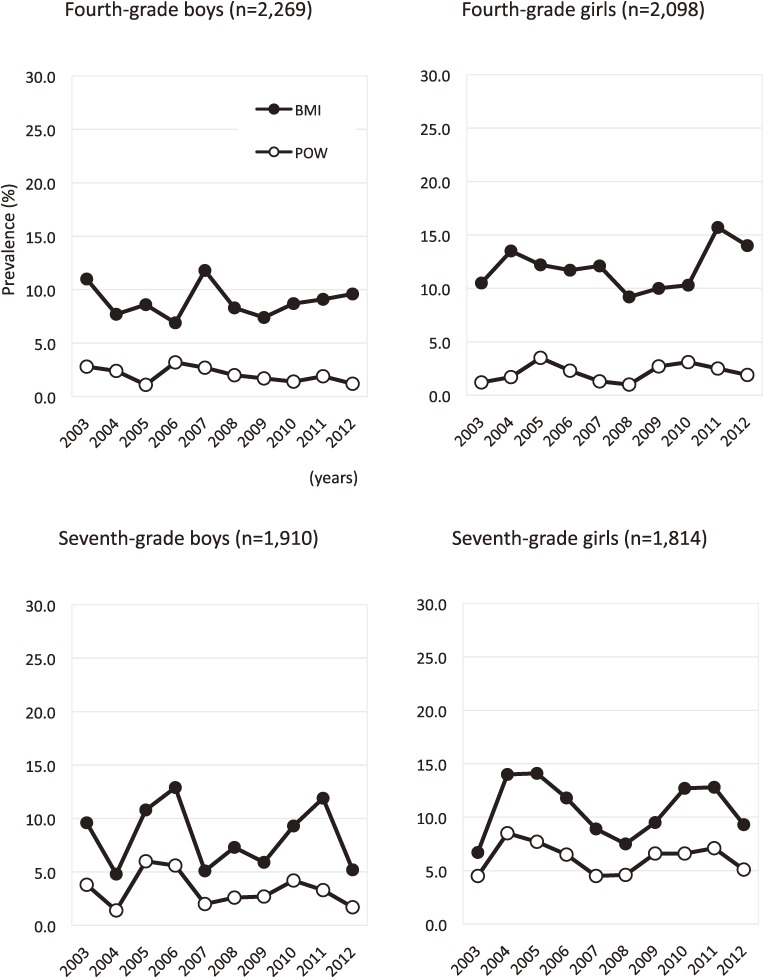
Trends in the prevalence of underweight by sex for each grade. BMI, body mass index as defined by the International Obesity Task Force. POW, percentage of overweight as defined by the Japanese Ministry of Education, Culture, Sports, Science and Technology.

The trends of underweight are shown in Figure 1. There were no significant changes in the prevalence of underweight from 2003 to 2012 regardless of grade, sex, and criteria, although there were pronounced change in the prevalence of underweight during some periods (for example, 2004–2005, 2006–2007, and 2011–2012 in seventh-grade boys). However, each year the prevalence of underweight defined by POW criteria was lower than that based on BMI criteria, regardless of grade and sex. Moreover, the trends in underweight using POW criteria were similar to those based on BMI criteria among schoolchildren.

**Figure 2.**
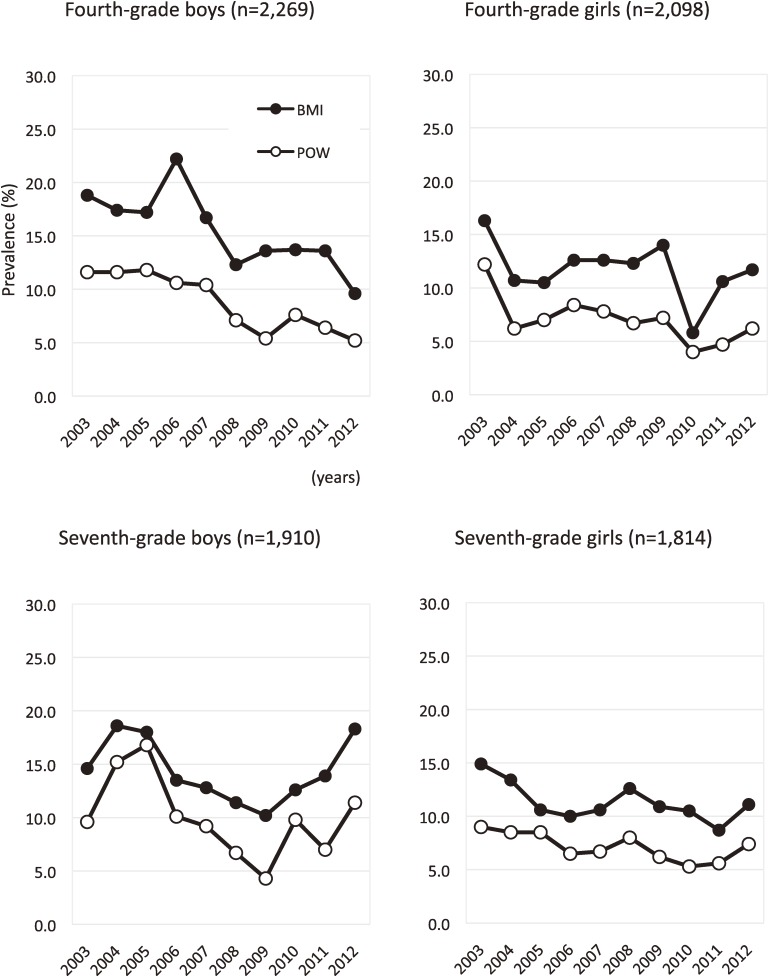
Trends in the prevalence of overweight by sex for each grade. BMI, body mass index as defined by the International Obesity Task Force. POW, percentage of overweight as defined by the Japanese Ministry of Education, Culture, Sports, Science and Technology.

The trends in overweight are shown in Figure 2. The prevalence of overweight according to both BMI and POW criteria decreased in fourth graders (BMI criteria: *P* = 0.006; y = −0.95x + 20.7 and POW criteria: *P* < 0.001; y = −0.81x + 13.2 in boys, *P* = 0.233; y = −0.37x + 13.8 and 0.028; y = −0.51x + 9.8 in girls, respectively). Among seventh-grade boys, the prevalence of overweight decreased from 2004 to 2009 but then increased, although the change in the prevalence of overweight boys during the study period was not significant. In contrast, the prevalence of overweight among seventh-grade girls significantly decreased between 2003 and 2012, regardless of criteria (BMI criteria: *P* = 0.047; y = −0.38x + 13.4 and POW criteria: *P* = 0.021; y = −0.30x + 8.8). In addition, each year the prevalence of overweight based on POW criteria was lower than that determined by BMI criteria, regardless of grade and sex, and the trends in overweight using POW criteria were similar to those based on BMI criteria.

## DISCUSSION

### Underweight

Using BMI criteria, the prevalence of underweight from 2003 to 2012 among fourth-grade boys and girls was 8.9% and 12.0%, respectively, while the prevalence among seventh-grade boys and girls was 8.3% and 10.7%, respectively. Using POW criteria, the prevalence of underweight was lower than that determined by BMI, regardless of sex and grade. In Japan, school health statistics calculated by MEXT using POW criteria show the nationwide prevalence of underweight and overweight children.^23^ Our study results suggest that the prevalence of underweight among Japanese schoolchildren as reported by MEXT has been underestimated, when compared to the prevalence of underweight according to BMI criteria.

In this study, there were no significant changes in the prevalence of underweight from 2003 to 2012, whereas the prevalence increased from 2009 to 2011. In Japan, the prevalence of underweight (BMI <18.5 kg/m^2^) and extreme underweight (BMI <17 kg/m^2^) among young girls (aged 15–19 years) has significantly increased according to nationwide data from the National Nutrition Survey.^24^ Moreover, a previous study in Japan reported that the desire for thinness has become more common even in pre-adolescent girls and, as a result, the tendency for pre-adolescent Japanese girls to develop eating disorders, such as anorexia or bulimia nervosa, is expected to grow in the future.^4^ Thus, distorted concern over body weight could increase the prevalence of underweight among Japanese schoolchildren, especially schoolgirls. Secular trends in underweight among children are predictors of future disease prevalence and should be assessed over longer periods of time.

### Overweight

Using the BMI cutoff points proposed by IOTF, the prevalence of overweight from 2003 to 2012 was 15.1% and 11.6% among fourth-grade boys and girls, respectively, and 14.3% and 11.2% among seventh-grade boys and girls, respectively. A recent study reported that the prevalence of overweight among sixth-grade boys and girls in Japan using IOTF BMI cutoff points was 20.6% and 14.9% in 2003 and was 16.4% and 12.0% in 2009.^18^ In our study, the prevalence of overweight from 2003 to 2004 was 18.1% and 13.5% among fourth-grade boys and girls, respectively, and 16.6% and 14.2% among seventh-grade boys and girls, respectively (data not shown); these results were similar to previous results.^18^ However, rates from 2009 to 2010 among fourth-grade boys and girls were 13.7% and 9.9%, respectively, and 11.4% and 10.7% among seventh-grade boys and girls, respectively (data not shown); our results were lower than previous results.^18^

In the present study, the prevalence of overweight based on POW criteria was lower than that based on BMI criteria, regardless of sex and grade, suggesting that the prevalence of overweight among Japanese schoolchildren, which has been determined using POW, has been underestimated. A previous study also reported that the governmental data for schoolchildren in Japan based on POW underestimated the current epidemic of obesity.^23^ However, Okuda et al showed that both BMI-based and POW-based cutoffs are useful for screening Japanese children and adolescents for obesity-related risk factors.^25^ Therefore, further studies are needed to determine which criteria should be used as a screening tool in Japan.

There was a decrease in the prevalence of overweight from 2003 to 2012 based on BMI criteria in our study. This decrease was found even when the POW definition of overweight was applied. A recent study showed that there was a slightly significant decrease in BMI and a decrease in the prevalence of overweight and obesity among schoolchildren in another area of Japan from 2003 to 2009,^18^ which is consistent with the results of the present study. This decreasing trend in the present study might be due to a health promotion program for the prevention of childhood lifestyle-related diseases being introduced in the study area. This program has enhanced parents’ and guardians’ awareness of obesity and childhood lifestyle-related disease, which could have helped reduce BMI among their children, thereby decreasing the prevalence of overweight.^17^^,^^21^ Although there was a decreasing trend in overweight from 2003 to 2009 in the present study, the prevalence of overweight increased from 2009 to 2012 among seventh-grade boys. Kouda et al reported an increase in obesity among boys from 1993 to 2008 in another area of Japan.^26^ Therefore, secular trends in prevalence of overweight among children should be closely monitored in the future in Japan.

### Strengths and limitations

To the best of our knowledge, this is the first report examining the prevalence and trends of underweight and overweight/obesity among population-based Japanese schoolchildren over the last decade using IOTF BMI cutoff points and POW in Japan. The participation rate in this study was high at over 99%, and data were obtained using a consistent measurement protocol from all children during their annual health checkups. However, there are some limitations in this study. First, we did not consider the lifestyle factors of the subjects. Several studies have shown the relationship between obesity or underweight in children and socioeconomic status,^14^^,^^15^^,^^19^ physical activity,^27^^,^^28^ and eating habits.^29^ Therefore, it will be necessary to consider these factors in future studies. Second, almost all of the subjects who were in the fourth grade from 2003 to 2009 in the present study were analyzed as seventh graders from 2006 to 2012. Therefore, the results for seventh graders could have been affected by the results for fourth graders, since many of the subjects were the same. Finally, since the subjects in this study were from only town-run schools in one town in Japan, the generalizability of the present findings to other populations might be limited.

### Conclusion

The present study showed that the prevalences of both underweight and overweight/obesity among Japanese schoolchildren as defined by POW criteria in Japan are underestimated compared to those determined by IOTF BMI cutoff points. The results of this study also suggest that trends in underweight and overweight/obesity using POW criteria are similar to those based on BMI criteria among schoolchildren in Japan.
